# EGCG reverses human neutrophil elastase-induced migration in A549 cells by directly binding to HNE and by regulating α1-AT

**DOI:** 10.1038/srep11494

**Published:** 2015-07-16

**Authors:** Yilixiati Xiaokaiti, Haoming Wu, Ya Chen, Haopeng Yang, Jianhui Duan, Xin Li, Yan Pan, Lu Tie, Liangren Zhang, Xuejun Li

**Affiliations:** 1 State Key Laboratory of Natural and Biomimetic Drugs, Peking University, Beijing 100191, China; 2 Department of Pharmacology, School of Basic Medical Science, Peking University and Institute of System Biomedicine, Peking University, Beijing 100191, China; 3 Department of Immunology, Peking University Health Science Center, Beijing 100191, China; 4 School of Pharmaceutical Sciences, Peking University, Beijing 100191, China

## Abstract

Lung carcinogenesis is a complex process that occurs in unregulated inflammatory environment. EGCG has been extensively investigated as a multi-targeting anti-tumor and anti-inflammatory compound. In this study, we demonstrated a novel mechanism by which EGCG reverses the neutrophil elastase-induced migration of A549 cells. We found that neutrophil elastase directly triggered human adenocarcinoma A549 cell migration and that EGCG suppressed the elevation of tumor cell migration induced by neutrophil elastase. We observed that EGCG directly binds to neutrophil elastase and inhibits its enzymatic activity based on the CDOCKER algorithm, MD stimulation by GROMACS, SPR assay and elastase enzymatic activity assay. As the natural inhibitor of neutrophil elastase, α1-antitrypsin is synthesized in tumor cells. We further demonstrated that the expression of α1-antitrypsin was up-regulated after EGCG treatment in neutrophil elastase-treated A549 cells. We preliminarily discovered that the EGCG-mediated induction of α1-antitrypsin expression might be correlated with the regulatory effect of EGCG on the PI3K/Akt pathway. Overall, our results suggest that EGCG ameliorates the neutrophil elastase-induced migration of A549 cells. The mechanism underlying this effect may include two processes: EGCG directly binds to neutrophil elastase and inhibits its enzymatic activity; EGCG enhances the expression of α1-antitrypsin by regulating the PI3K/AKT pathway.

Of the wide variety of cancer types, lung cancer is the first and the second leading cause of cancer death globally in males and females, respectively. The 5-year survival rate of lung cancer is only approximately 15% despite therapeutic advances in its diagnosis and treatment in recent decades^1^. The surrounding inflammation in the tumor microenvironment exerts many tumor-promoting effects that accelerate the proliferation, migration and survival of malignant cells and that promote angiogenesis and metastasis^2^. An improved understanding of the molecular mechanisms involved in tumor-associated inflammation may provide additional insight into the diagnosis of lung cancer and the development of drugs for its treatment.

(−)-Epigallocatechin-3-gallate (EGCG), the most abundant and biologically active catechin, is well known to induce proliferation and apoptosis in many types of cancer, such as lung cancer^3^, pancreatic cancer^4^, and colon cancer^5^,^6^,^7^. Regarding its mechanisms of action, previous studies have shown that EGCG exerts its anti-tumor effects via the regulation of various protein kinases, including mitogen-activated protein kinase (MAPK) family members, especially Akt kinases^8^. EGCG regulates various major signaling pathways, such as the activator protein (AP), nuclear factor-κB (NF-κB), and phosphatidylinostitol–3-OH kinase pathways (PI3K/Akt)^5^,^6^,^7^. However, the molecular mechanisms underlying the anti-tumor metastatic properties of EGCG in the specific context of inflammation remain elusive. In this study, we demonstrated additional contributions of inflammation to the progression of lung cancer metastasis and a novel molecular target of EGCG, human neutrophil elastase, which induces lung cancer cell migration.

Neutrophils, as a component of the tumor microenvironment, have only recently been thought to play an important role in tumor growth and invasiveness^9^. The presence of increased infiltrated neutrophils within tumors was significantly associated with a poorer clinical outcome in patients with bronchioloalveolar carcinoma^10^. Neutrophil elastase, which is released by activated neutrophils, is the most potent neutrophil protease. The potential substrates of this protease include cytokines and cytokine receptors, indicating that neutrophil elastase may regulate the inflammatory process^11^. Recent reports have shown that neutrophil elastase directly induces uncontrolled tumor proliferation in a lung adenocarcinoma mouse model and in lung epithelial tumor cells.

Alpha-1 antitrypsin (α1-AT, AAT) is a serum glycoprotein that contains three potential glycosylation sites. As an acute-phase protein, AAT is thought to play an important role in limiting host tissue injury triggered by proteases, particularly human neutrophil elastase (HNE). The clinical relevance of AAT is demonstrated in individuals with an inherited deficiency in circulating AAT, who exhibit increased susceptibility to early-onset pulmonary emphysema and liver and pancreatic diseases^12^. Neutrophil elastase and AAT are a protease and protease inhibitor counterpart pair^13^. It has been assumed that in AAT-deficiency, the protease/ anti-protease balance is shifted toward HNE, which leads to extensive tissue damage, particularly by causing emphysema. Xu *et al.*^14^ demonstrated that the natural polyphenol product curcumin inhibits tumor proliferation induced by neutrophil elastase via the upregulation of AAT in lung cancer. Altering the balance between AAT and HNE may represent an innovative form of lung cancer treatment.

## Materials and methods

### Cell culture and treatment

The human lung adenocarcinoma cell line A549 was used for *in vitro* experiments. The cells were maintained in Dulbecco’s modified Eagle’s medium (DMEM, Gibco, NY, USA) containing 10% fetal bovine serum (FBS), 100 U/ml penicillin and 100 μg/ml streptomycin in a humidified incubator containing 5% CO_2_ in air at 37 °C. The cells were treated with different concentrations of neutrophil elastase (Merck Group, Darmstadt, Germany) and/or EGCG (Sigma-Aldrich Co., St. Louis, MO, USA) for 24 h and then evaluated as described below.

### Cell viability assay

Cell viability was determined using a Cell Titer 96 ^®^ Aqueous One Solution Cell Proliferation Assay (MTS assay, Promega, Madison, WI, USA). A549 cells were seeded on 96-well plates at a density of 5000 cells/well and were allowed to adhere for 36 h. After incubation in neutrophil elastase and/or EGCG for 24 h, 10 μl of the MTS solution was added to each well, followed by incubation for 2 h at 37 °C. The resulting color was assayed at 490 nm using a microplate reader (Bio-Rad Laboratories, Inc., Hercules, CA, USA).

### *In vitro* wound healing assay

The wound healing assay was performed as previously described (Liang *et al.*, 2007) to assess the capacity for cell migration. Briefly, the A549 cells were seeded on a 6-well plate. When the cells reached 90–95% confluence, scratches were applied using a sterile 200-μl pipette tip. The cells were washed three times with phosphate buffered saline (PBS), and the initial wounds were examined under an Olympus microscope. Then, the cells were incubated in medium containing neutrophil elastase and/or EGCG for 24 h, and the wounds were photographed again. The rate of cell migration was evaluated based on the rate of wound closure.

### Transwell migration assay

Cell migration was further investigated using the transwell migration assay. A549 cells were suspended in serum-free DMEM (1×10^5^ cells) and placed in the upper chamber. The plate was incubated at 37 °C for 18 h, followed by fixation of the cells in 4% formaldehyde. The upper chamber was gently wiped with a cotton swab to remove the non-migrated cells, and the migrated cells on the underside of the polycarbonate filter were stained with crystal violet and counted under an Olympus microscope (×200 magnification).

### Immunofluorescence assay

A549 cells were seeded on glass coverslips in an environment containing 5% CO_2_ in air at 37 °C and then incubated in 10 nM neutrophil elastase and 20 μM EGCG for an additional 24 h. The coverslips were gently washed in PBS, fixed in 3.7% paraformaldehyde for 15 min, permeabilized in PBS containing 0.1% Triton X-100 for 20 min and blocked in PBS containing 3% BSA for 30 min. The fixed cells were incubated in an anti-human zinc finger E-box-binding protein-1 (ZEB-1) antibody (Cell Signaling Technology, Beverly, MA, USA) overnight at 4 °C in a humidified chamber. Then, the cells were washed and incubated in the corresponding DyLight 488-conjugated secondary antibody (Jackson ImmunoResearch Laboratories, West Grove, PA, USA) for 1 h at 37 °C. After washing in PBS, the cells were incubated in Hoechst 33342 (1 μg/ml, Sigma-Aldrich) for 5 min at room temperature. After several washes, the immunostained specimens were observed under a TCS-SP5 laser scanning confocal microscope (Leica Microsystems, Wetzlar, Germany).

### Molecular docking and molecular dynamics

The CDOCKER algorithm in Discovery Studio (DS) 2.5 (Accelrys Software, Inc., San Diego, CA) using the CHARMm engine was used in this study to evaluate the potential molecular binding mode between EGCG and HNE. The program was run using a Dell Optiplex755 server (Round Rock, TX). The CDOCKER algorithm employed a strategy involving the generation of the initial ligand orientation in the active site of the target protein followed by molecular dynamics (MD)-based simulated annealing and final refinement via energy minimization. The crystal structure of HNE (PDB ID: 2Z7F) was obtained from the Protein Data Bank (http://www.rcsb.org/pdb). The water molecules in the protein were removed, the protein was refined based on the root mean square deviation (RMSD), and EGCG was docked to the protein according to the appropriate parameters. Beginning from the EGCG configuration, a set of 10 different orientations was randomly generated. The co-crystalized ligand SEI300 was used as the positive control ligand, and the binding site was defined based on the binding of SEI300 and HNE. The interaction energy was calculated to analyze the interaction between the ligand and the receptor. Once the ligand was docked to the active site, a molecular dynamics (MD) simulation was performed using the GROMACS 4.6.6 program. The GROMOS96 53a6 Force Field and the SPC/E water condition were selected during the topology process. Water was position-restrained using a force constant (kpr) of 1000 kJ mol^−1^·nm^−2^. The protein was placed in the box at least 1.0 nm from the box edge, and the box was defined as a cube. During the GENION process, 10 Cl^−1^ were added to the system for charge equilibration. A 30-ns MD simulation was performed. The GROMACS program was used to constrain the bonds involving hydrogen atoms, allowing for an integration interval of 2 fs. The particle mesh Ewald method using a threshold of 1 nm was employed to account for the electrostatic interactions. A threshold of 1 4 nm was used for the van der Waals interactions. The non-bonded pair lists were updated every 0.010 ps. The coordinates were saved every 1 ps. Accuracy testing was performed by calculating the RMSD after re-docking the internal ligand to the protein using the algorithm.

### Binding free energy calculations

The trajectories obtained from molecular dynamics were used for binding free energy calculations. The calculations were performed using GROMACS Molecular Mechanics Poisson Boltzmann Surface Area (g_mmpbsa) method, implemented in GROMACS 4.6.6^15^ The binding free energy for a protein–ligand complex is given as:


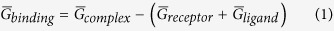


where, *△G*_*binding*_ is an estimate of absolute free energy of binding and 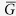 is the average free energy of complex, receptor and ligand respectively. The average free energy on the other hand is defined as:


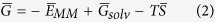


Where, 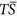 is the solute entropic contribution to the system at temperature *T*. 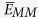 is the molecular mechanical energy obtained from bonded and non-bonded interactions within the system and can be represented by following equation:


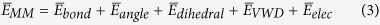



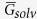
 is the average solvation free energy and is equal to the sum of electrostatic and non-polar terms and represented as:


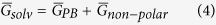



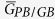
 is the electrostatic contributions to solvation free energy which is evaluated by Finite Differential Poisson Boltzmann (FDPB) in case of *PBSA* method. 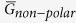 is the hydrophobic non polar contributions to solvation free energy, calculated as:


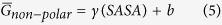


Where *SASA* is the solvent accessible surface area and, 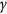 and b are constants.

For the *g_mmpbsa* calculations the molecular dynamic trajectories were split into “snapshots”. A total 1,000 snapshots were extracted from 20,000 frames obtained in 10 ns of production run. This allows not only to sample the flexibility of the binding site, but also to obtain a more reliable free energy estimate of binding than compared to a single snapshot calculation.

### Surface plasmon resonance (SPR) biosensor analysis

The binding affinity of EGCG to HNE *in vitro* was assayed using the SPR-based Biacore 3000 instrument (Biacore AB, Uppsala, Sweden) as previously reported^16^,^17^. Recombinant HNE protein (molecular mass, 25 kDa; Pl 5.1 in PBS) with a purity of greater than 95% was purchased from Merck Group (Darmstadt, Germany). The manufacturer indicated that this material could be used for *in vitro* binding assays. The HNE protein was dissolved in coupling buffer (15 μg/ml, in 10 mM sodium acetate, PH 4.50), and 412 response units (RU) of the HNE protein, which was used as the ligand, were immobilized on a CM5 sensor chip using N-ethyl-N-(3-dimethylaminopropyl) carbodiimide and N-hydroxysuccinimide according to the standard primary amine-coupling procedures; HBS-EP (10 mM HEPES, 150 nM NaCl, 3 mM EDTA, and 0.005% (v/v) surfactant P20, pH 7.4) was used as the running buffer. Equilibration of the baseline was performed by continuously flowing HBS-EP across the chip surface for 1–2 h. The Biacore data were collected at 25 °C using HBS-EP as the running buffer at a constant flow rate of 30 μl/min. EGCG was serially diluted into the running buffer to create a series of concentrations. The samples were injected into the channels at a flow rate of 30 μl/min, followed by washing with running buffer. The binding responses were continuously recorded in RU at a frequency of 1 HZ as sensorgrams and were expressed as a function of time. The association (K_on_) and dissociation (K_off_) rate constants and the equilibrium dissociation constant (K_D_= K_off_) were calculated as concentrations of EGCG using BIA evaluation software version 3.1 (Biacore) and the 1:1 Langmuir binding fitting model. The curve-fitting efficiency was evaluated according to the statistical parameter χ^2^.

### Western blot analysis

Total protein was extracted using RIPA lysis buffer, and equal amounts of proteins were subjected to 8% SDS-PAGE and then transferred to polyvinylidene difluoride membranes (Millipore Corp., MA, U.S.A). The membranes were blocked and then incubated in primary antibodies against vimentin, β-catenin, AAT), insulin receptor substrate-1 (IRS-1) (1:1000, Cell Signaling Technology, Beverly, MA), pAkt and Akt (1:1000, Cell Signaling Technology), pPI3K and PI3K (1:1000, Cell Signaling Technology), and GAPDH (1:10,000 Sigma-Aldrich) overnight at 4 °C with gentle agitation, followed by incubation in the anti-rabbit IR-Dye 670- or 800cw-labeled secondary antibody for 1 h at room temperature. Two 10-min washes in TBST were performed after secondary antibody labeling; then, the membranes were placed in TBST. The membranes were imaged using a LiCor Odyssey scanner. Boxes were manually placed surrounding each band of interest to measure the raw near-infrared fluorescence intensity values, and the intra-lane background intensity was subtracted using Odyssey 3.0 analytical software (LiCor, Lincoln, NE, USA).

### HNE activity assay

The assessment of HNE activity was performed according to Löser^18^. Briefly, 125 μl of substrate solution (1.4 mM N-MeO-Suc-Ala-Pro-Val-p-NA in Tris-HCl-buffer, pH 7.5) was added to 405 μl of Tris-HCl buffer, pH 7.5, and 50 μl of extract solution. After the addition of 20 μl of enzyme solution (approximately 3 mU), the samples were vortexed and then incubated for 1 h at 37 °C. The reaction was terminated via the addition of 500 μl of soybean trypsin inhibitor solution (0.2 mg/ml in Tris-HCl buffer, pH 7.5). The samples were then vortexed, and the absorbance was measured at 405 nm. The remaining activity of HNE (as a % of the control) was calculated relative the control in the absence of the inhibitor, considering the influence of the buffer, the substrate, the solvent and the extract. The inhibitory effect of the selective HNE inhibitor sivelestat sodium (4 μmol/L), which was previously established using the same assay, was considered as a positive control.

## Results

### EGCG inhibits tumor cell migration induced by neutrophil elastase but does not influence the proliferation of A549 cells

**Figure 1:**
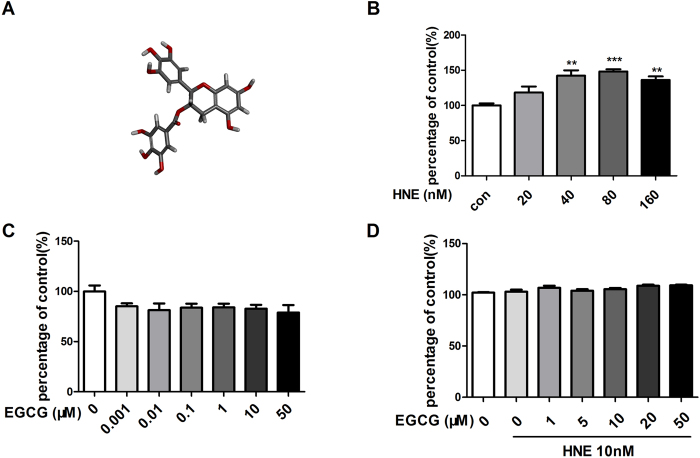
Effects of EGCG on HNE-induced A549 cell viability. (**A**)Molecular structure of EGCG. (**B**) HNE promoted the viability of NSCLC A549 cells. The cells treated with the indicated concentrations of HNE for 24 h were assessed for viability via the MTS assay. The data represent the means ± SEM, n = 3. **P < 0.01, ***P < 0.001 *vs* the non-HNE-treated group (control). (**C**) EGCG did not affect the viability of A549 cells. The cells treated with the indicated concentrations of EGCG for 24 h were assessed for viability using the MTS assay. (**D**) EGCG did not affect the viability of the HNE (10 nM)-induced A549 cells.

**Figure 2:**
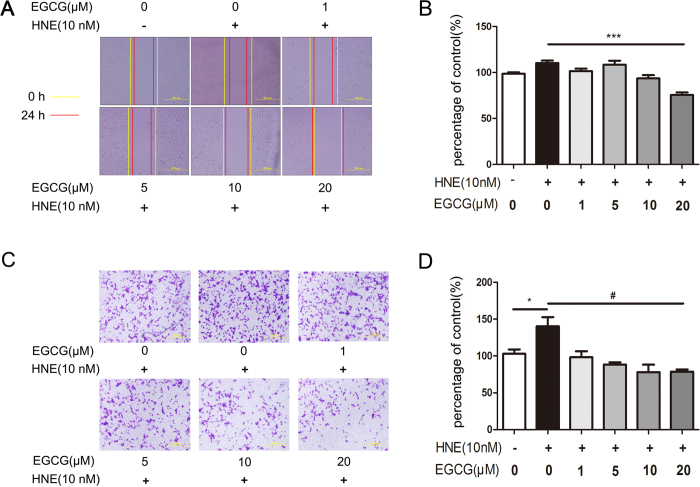
EGCG ameliorated the HNE-induced migration of A549 cells. (**A**) After wounding, the **A**549 cells were treated with HNE in the presence or absence of the indicated concentrations of EGCG for 24 h; cell migration was then assessed by measuring the rate of wound healing for each scratch, in which 6 different visual fields were examined for each measurement. The images show the groups treated with blank solvent (control) or HNE (10 nM) in the presence or absence of the indicated concentrations of EGCG. The lines indicate the wound edges (yellow, 0 h; red, after 24 h). The scale bar indicates 500 μM. (**B**) The statistical results for all treatment groups in the wound healing assay. (**C**) The cells were seeded in a transwell chamber and treated with the indicated concentrations of EGCG with or without HNE (10 nM). The number of migrated cells per field was counted in 12 visual fields. Representative visual fields of the groups treated with EGCG at the indicated concentrations with or without HNE (10 nM) are shown. The scale bar indicates 500 μm. (**D**) The statistical results for all treatment groups in the transwell migration assay. The results represent the means ± S.E.M. of triplicate experiments. *P < 0.05 and ***P < 0.001: significantly different from the control group based on the two-tailed Student’s *t*-test. # P < 0.05: significantly different from the HNE (10 nM)-treated group based on the two-tailed Student’s *t*-test.

To evaluate the anti-metastatic effect of EGCG on lung cancer with neutrophil elastase involvement *in vitro*, we first treated the metastatic A549 cells with various concentrations of neutrophil elastase (20–160 nM), EGCG (up to 50 μM) or EGCG (up to 50 μM) and neutrophil elastase (10 nM). The results from the MTS proliferation assay showed that neutrophil elastase at a concentration of at least 20 nM enhanced tumor cell viability; among all of the concentrations tested, 40 nM neutrophil elastase displayed the strongest effect (Fig. 1B). Thus, we selected 10 nM neutrophil elastase as the intervention condition. The MTS proliferation assay results showed similar cell viability between the control group and the groups treated with various concentrations of EGCG (Fig. 1C) and also showed a non-significant change in cell viability between the group treated with neutrophil elastase (10 nM) and the groups treated with EGCG and neutrophil elastase (10 nM). However, in the wound healing assays, the cells treated with 10 or 20 μM EGCG migrated at a much slower rate than the control cells and the cells treated with neutrophil elastase (10 nM) (Fig. 2A,B), suggesting that EGCG displays anti-migratory properties. To confirm this result, we performed further cell migration assays using the transwell chamber model. As shown in Fig. 2C,D, the group treated with 10 nM neutrophil elastase displayed significantly more migrating A549 cells than the control group. Alternatively, treatment with either 10 or 20 μM EGCG (after exposure to 10 nM neutrophil elastase), resulted in dramatically fewer migrating A549 cells than the control treatment and treatment with neutrophil elastase (10 nM) alone. These results indicated that treatment with EGCG at a concentration between 10 and 20 μM induces a substantial anti-migratory effect without affecting the proliferation of A549 cells exposed to neutrophil elastase.

### EGCG inhibits the HNE-induced regulation of epithelial-mesenchymal transition (EMT) markers.

**Figure 3:**
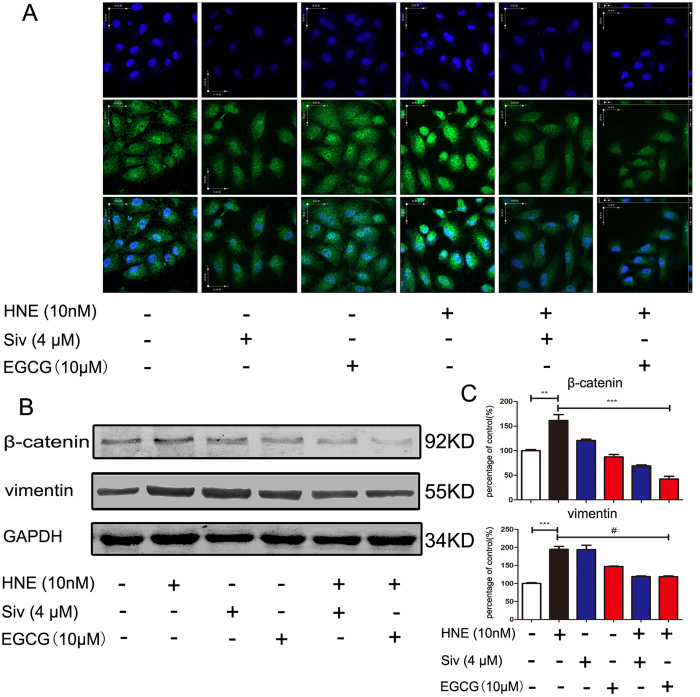
EGCG regulated the HNE-induced change in the expression of EMT markers. (**A**) Confocal images of ZEB-1 in A549 cells exposed to EGCG, sivelestat sodium and neutrophil elastase. A549 cells were grown to 60% confluence and then incubated in 20 μM EGCG, 4 μM sivelestat sodium and/or 10 nM neutrophil elastase for 24 h. The cells were labeled with an anti-human ZEB-1 antibody, a DyLight 488-conjugated secondary antibody (green) and the Hoechst 33342 stain (blue). (**B**, **C**) The protein levels of vimentin were clearly down-regulated in response to EGCG treatment with or without HNE. (**B**, **D**) The expression of β-catenin was increased after HNE induction but was significantly decreased after treatment with 20 μmol/L EGCG. The gels were electrophoresed using the identical experimental conditions.

To determine whether EGCG regulates the HNE-induced EMT process, the expression of EMT marker proteins was detected via western blot assays and confocal microscopy. The results showed that vimentin expression was significantly altered after HNE treatment. However, as the specific inhibitor of HNE sivelestat sodium significantly up-regulated the expression of vimentin. Nevertheless, the vimentin protein levels were clearly down-regulated in response to EGCG treatment in the presence or absence of HNE (Fig. 3B,C). The expression of β-catenin was increased after HNE induction but was significantly decreased after treatment with 20 μmol/L EGCG (Fig. 3B,C). We also detected the expression and sub-cellular localization of ZEB-1 because a recent study suggested that ZEB-1 plays a role in lung cancer invasiveness and metastasis development. The results showed that ZEB-1 expression was significantly increased after treatment with HNE, and this effect was ameliorated after the addition of EGCG (Fig. 3A). Additionally, the sub-cellular translocation of ZEB-1 from the cytoplasm to the nucleus was detected after treatment with HNE. The expression of ZEB-1 in the nucleus was decreased after treatment with EGCG in the neutrophil elastase-treated A549 cells.

### Identification of the interaction between EGCG and HNE

**Figure 4:**
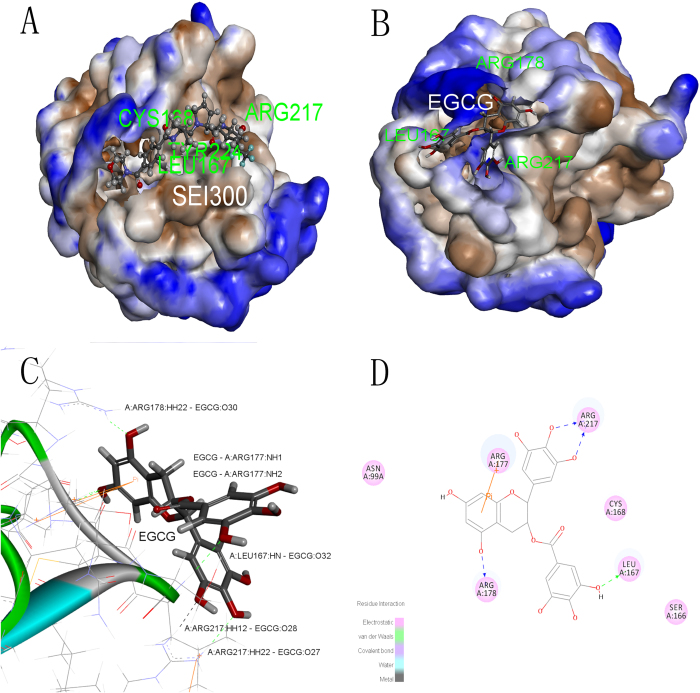
Illustration of the docking of the EGCG molecule to the HNE protein based on the CDOCKER algorithm (Discovery Studio 2.55). The endogenous ligand SEI300 (**A**) and EGCG (**B**) molecule is displayed using a ball and stick model and the protein is displayed using a macromolecular surface model; the detailed interaction between EGCG and HNE (**C**); 2D-interaction analysis between EGCG and HNE (**D**).

**Figure 5:**
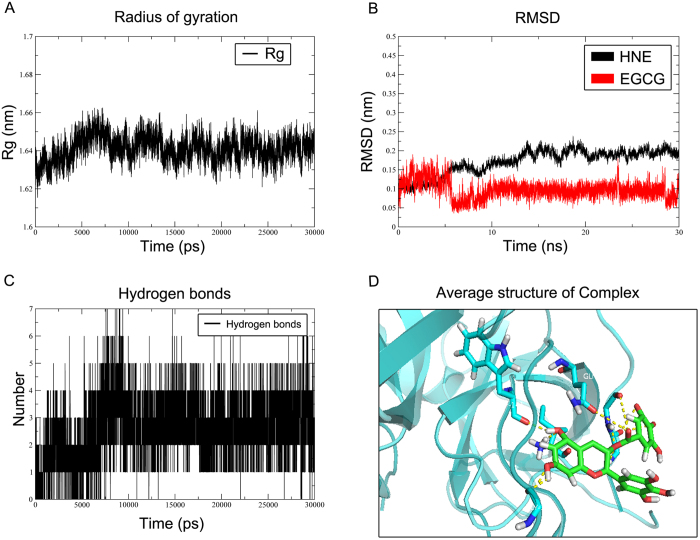
MD process using the GROMACS 4.6.6 simulation protocol. The time-averaged normalized ratio of the gyration radius (**A**), the RMSD (**B**), the number of hydrogen bonds (**C**) and the average structure of EGCG-HNE complex from 20 nm to 30 nm (**D**) were analyzed to reflect the distribution of EGCG molecules surrounding HNE.

We analyzed the binding pattern between EGCG and HNE. First, a crystal structure of HNE bound to a selective inhibitor (PDB ID: 2Z7F) was selected. The interaction energy between EGCG and HNE is –29.9704 kJ/mol, which is similar to the interaction energy (−34.0679 kJ/mol) between HNE and its endogenous inhibitor SEI300. The active binding site between EGCG and HNE (PDB ID: 2Z7F) was defined as the ligand-binding site with a 9-Å radius. As anticipated, EGCG stably docked to the ligand-binding domain (LBD) (Fig. 4B). At least 8 residues in the LBD were involved in the interaction between EGCG and the HNE protein. EGCG potentially formed hydrogen bonds with SER166, THR165, ARG217 and ARG178. Moreover, EGCG possessed a potential aromatic interaction with ARG177 (Fig. 4D). The binding mode of EGCG in the LBD of HNE provided detailed structural insight into the interaction between this compound and the HNE protein. Furthermore, we performed a 30-ns MD process using GROMACS 4.6.6 software. The time-averaged normalized ratio of the gyration radius, the RMSD, hydrogen bonds and the GROMACS energy were analyzed to reflect the distribution of EGCG molecules surrounding HNE. The gyration radius and the RMSD of the ligand EGCG and the receptor HNE trended toward stability as time progressed (Fig. 5A,B). The interacting H-bond number was shown to change with in a stable range as time progressed (Fig. 5C). Additionally, the GROMACS energy did not fluctuate as time progressed changed (Fig. 5D). g_mmpbsa method calculated results showed that ∆*G*_*binding *_= −71.8 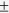 12.4.KJ/mol (Table 1).

### The binding affinity of EGCG to HNE based on SPR biosensor analysis

**Figure 6:**
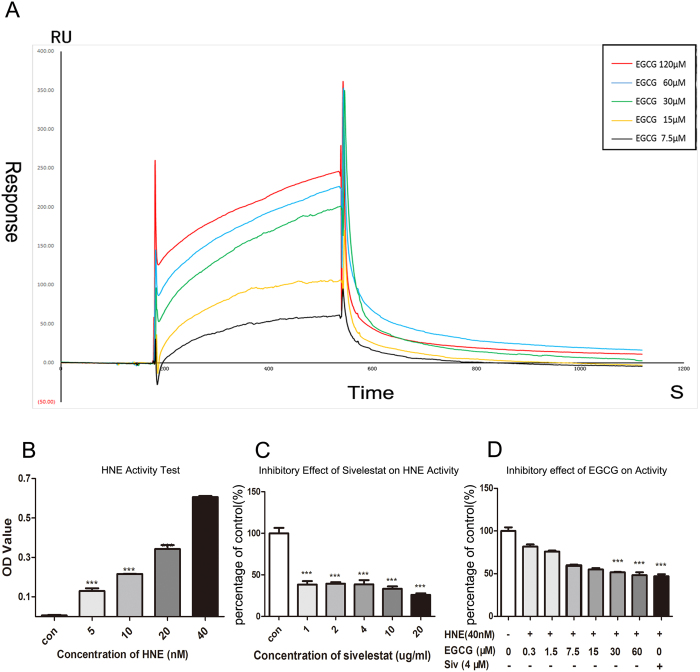
The binding affinity of EGCG to neutrophil elastase as determined by SPR, and the inhibitory effect of EGCG on neutrophil elastase activity as determined by an enzymatic activity assay. (**A**) Real-time measurements of the binding affinity of EGCG to neutrophil elastase were outperformed using a Biacore 3000 instrument. Then, representative sensorgrams were obtained by injecting EGCG at a concentration of 7.5, 15, 30, 60 or 120 μM (curves from bottom to top) above the immobilized surface covered with neutrophil elastase. EGCG was injected for 30 s, and dissociation was monitored for more than 120 s. (**B**) Verification of the *in vitro* activity of neutrophil elastase. (**C**) Application of the neutrophil elastase inhibitor sivelestat sodium as a positive control. (**D**) The enzymatic activity of neutrophil elastase was decreased after EGCG treatment in a concentration-dependent manner.

To verify the prediction from the CDOCKER-based analysis that EGCG directly binds to the HNE protein, the binding affinity of EGCG to HNE was determined using SPR biosensor technology. The ability of EGCG to bind to HNE was reflected by the RU values that were directly recorded from the Biacore 3000 instrument. As shown in Fig. 6A, the RU increased with increasing EGCG concentration, indicating that EGCG bound to HNE in a concentration-dependent manner. The association (k_on_), dissociation (K_off_), and equilibrium dissociation constants (K_D_) of EGCG binding to HNE were 98.6 × 10^3^ M^−1^·S^−1^, 4.08 × 10^−3^ S^−1^, and 4.14 × 10^−5^ M, respectively. The curve-fitting efficiency was evaluated according to the Χ^2^, a statistical parameter in the SPR assay. The Χ^2^ value was calculated to be 6.94. The results indicated that EGCG displayed specific binding affinity for HNE (Fig. 6A).

### EGCG inhibits the enzymatic activity of HNE

We first tested the enzyme activity of HNE by adding the indicated concentrations of HNE to the substrate solution system. The results showed that HNE reacted with the substrate in a concentration-dependent manner (Fig. 6B). After establishing the steady enzyme-substrate reaction system, we tested the inhibitory effect of EGCG at the indicated concentrations. Sivelestat sodium was used as the positive control to evaluate the inhibitory effect of EGCG. The results showed that EGCG inhibited HNE activity in a concentration-dependent manner; the IC50 of EGCG was 27.34 μM (Fig. 6C), which was higher than that of sivelestat sodium (IC50 = 3.62 μM) (Fig. 6D).

### EGCG ameliorates the HNE-induced expression of AAT and IRS-1 via the Akt/PI3K signaling pathway

**Figure 7:**
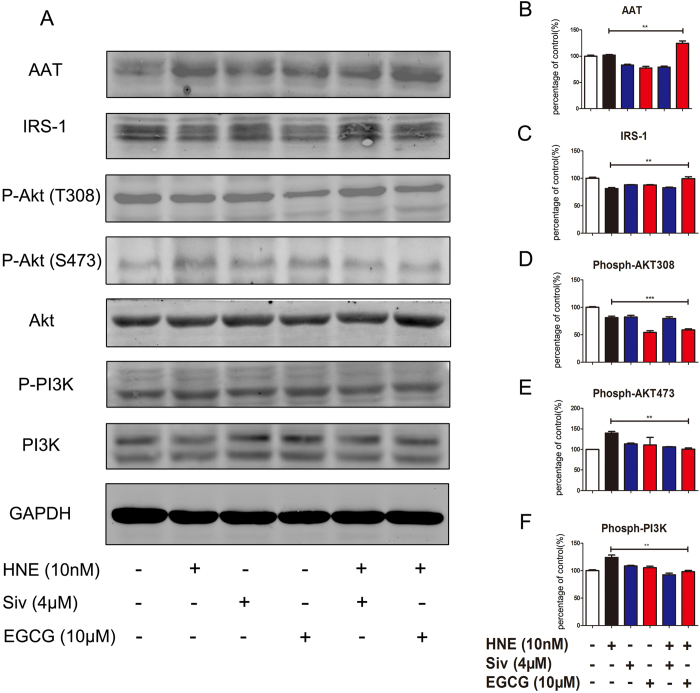
EGCG attenuates the neutrophil elastase-induced cell migration via the PI3K/Akt pathway. (**A**) Western blot for AAT, IRS-1, pAkt (phosphorylated at Thy308 or Ser473), Akt, p-PI3K, PI3K and GAPDH in A549 cells treated with EGCG or sivelestat sodium with or without HNE. (**B**-**F**) The statistical data are presented as histograms of the Western blot results for AAT (B), IRS-1 (**C**), the pAkt (Thy308)/Akt ratio (**D**), the pAkt (Ser473)/Akt ratio (**E**) and the pPI3K/PI3K ratio (**F**) in A549 cells treated with neutrophil elastase, EGCG and sivelestat sodium. The data are presented as the means ± SEM; n = 3. **P < 0.01; ***P < 0.001. The images for IRS-1, AAT and GAPDH were cropped from the same gel. The images for p-PI3K, p-Akt (T308) and GAPDH were cropped from the same gel. The images for PI3K and p-Akt (S473) were cropped from the same gel. The images for Akt and GAPDH were cropped from the same gel. All gels were electrophoresed using the identical experimental conditions.

AAT is a serine protease inhibitor (SERPIN) and is the natural inhibitor of HNE. The imbalance between AAT and HNE plays an important role in lung cancer progression^13^. Based on this understanding, we predominantly focused on the regulation of AAT by EGCG to investigate the mechanisms by which EGCG inhibits HNE-induced cell proliferation. Western blot assays were used to assess whether the AAT protein levels were altered by EGCG in A549 cells. When co-incubated with neutrophil elastase (10 nM), EGCG (20 μM) enhanced AAT expression in A549 cells (Fig. 7A,B). Neutrophil elastase directly induced lung tumor cell proliferation by degrading IRS-1, which is an adapter protein of PI3K, and subsequently activating the PI3K pathway in the tumor cells^19^. Upon neutrophil elastase exposure, the protein level of IRS-1 was decreased, and the phosphorylation of Akt (Thr 473) and PI3K were elevated. EGCG (20 μM) enhanced the protein levels of IRS-1 (Fig. 7A,C) and reduced the phosphorylation of Akt and PI3K in the neutrophil elastase-stimulated A549 cells (Fig. 7A,D–F).

## Discussion

In recent years, EGCG has been reported to inhibit tumor proliferation and metastasis and to induce the apoptosis of lung cancer cells *in vitro* and *in vivo*^20^. EGCG suppresses proinflammatory cytokines and chemokines induced by Toll-like receptor 9 agonists in prostate cancer cells^21^. In addition, EGCG suppresses lung cancer cell growth via Ras-GTPase-activating protein SH3 domain-binding protein-1^22^. Many molecules are involved in the anti-tumor activity of EGCG, including JAK/STAT, MAPK, PI3K/Akt, Wnt, Notch, NF-κB and AP-1^23^. Because cancer progression is a complex process, its pathogenic mechanism remains somewhat elusive. EGCG is considered as a natural multi-targeting chemopreventive agent. Thus, the possibility that EGCG inhibits tumor development via alternative mechanisms cannot be excluded. The purpose of this study was to investigate the novel inflammation-related mechanisms by which EGCG mediates anti-cancer migratory activity in lung cancer.

Recent studies suggested that chronic inflammatory pulmonary diseases such as emphysema are highly associated with an increased risk of lung cancer, independent of smoking^24^. HNE, among the most potent proteinases released by neutrophils, is considered to be responsible for the elastolytic damage in emphysema^25^. To some extent, neutrophil elastase may represent the link between emphysema and lung cancer. Recent reports showed that modest levels of neutrophil elastase led to the uncontrolled proliferation of A549 lung epithelial tumor cells^19^. We found that HNE treatment (10 nM) enhanced the migration of A549 cells. The concentrations of neutrophil elastase used in our study were lower than 40 nM to avoid the proliferation-promoting effect of HNE. Our results indicated that 20 μM EGCG completely suppressed the enhancement of cell migration induced by HNE in A549 cells. Furthermore, we verified that EGCG ameliorated the neutrophil elastase-induced up-regulation of the expression of several EMT markers, such as ZEB-1 and vimentin. Notably, it was recently reported that tumor-entrained neutrophils (TENs) induced by a primary tumor accumulated in the circulation and the premetastatic lung and inhibited the metastatic seeding of tumor cells in the lungs by generating H_2_O_2_ in mouse models of breast cancer^26^. Therefore, the role of HNE, which is generally considered as the major effector of human neutrophil function, particularly merits investigation. Thus, we provide the first evidence for the proliferation-promoting effect of HNE on A549 cells.

Next, we explored whether EGCG directly binds to HNE using the CDOCKER algorithm. CDOCKER is an efficient technique used to predict interactions between small molecules and proteins by modeling and RMSD analysis. The binding site was defined based on the definition of the positive control ligand (SEI300) binding site. The analysis showed that ARG A: 217 and THR A: 165 formed hydrogen bonds with the hydrogens on the phenolic hydroxyl groups of the small molecule EGCG. Additionally, ARG A: 177 formed a Pi bond with EGCG.

Applying minimization and MD to EGCG and HNE enabled us to perform a ligand-protein interaction analysis, as depicted in Fig. 5A–D. To determine the specific binding site, we directly transformed the CDOCKER binding results in the PDB file to an MD preparation GRO file. This analysis showed that the interaction between EGCG and HNE may not involve instantaneous binding but rather may be a long term interaction. Based on the results of MD simulation, we further calculated the binding free energy. According to study of Sulea *et al.*^27^, we verified that EGCG could bind with HNE protein. To verify these results concerning the interaction between EGCG and HNE, we further evaluated the binding affinity of EGCG to HNE using SPR biosensor analysis. The results confirmed the computer modeling prediction. We are the first to discover this novel target of the multi-targeting molecule EGCG. Interestingly, the inhibitor of HNE sivelestat sodium displayed a lower affinity to HNE than EGCG. We found that the low water solubility of sivelestat sodium led to the appearance of the lipid solvent as an interfering factor. Because of equipment limitations, we could not resolve this issue in the present study. To confirm the inhibitory effect of the interaction between EGCG and neutrophil elastase on the activity of this enzyme, we measured the enzymatic activity of neutrophil elastase after treatment with EGCG or sivelestat sodium. Alasbahi^28^ had verified the method of the elastase enzymatic activity assay. EGCG exerted a concentration-dependent inhibitory effect on the enzymatic activity of neutrophil elastase. As the positive control, sivelestat sodium exerted a greater inhibitory effect than EGCG.

As AAT is the specific and primary inhibitor of neutrophil elastase, we assumed that AAT might mediate the inhibitory effect of EGCG on neutrophil elastase-induced cell migration *in vitro*. AAT is predominantly synthesized in liver but is also expressed in extra-hepatic tissues and cells, including carcinoma cells^29^. The role of AAT in cancer development has been supported by some laboratory data; however, the molecular mechanism underlying the role of AAT in tumor cell migration is poorly understood. The primary function of AAT is the inhibition of neutrophil elastase. Our results indicated that the protein levels of AAT in A549 cells were clearly increased after treatment with EGCG. However, the contribution of AAT to the tumor development is somewhat controversial. In contrast to its effects against tumor growth, AAT has been shown to be correlated with poor prognosis in lung adenocarcinoma^30^ and with the inhibitory effect of polymorphonuclear neutrophils (PMNs) on the proliferation and invasiveness of lung cancer HCC cells^31^. Upon binding to neutrophil elastase, AAT activates cleavage within the reactive site loop (RSL) in a suicidal action^32^. Evidence has indicated that the AAT levels are significantly elevated in the skin of NE^−/−^ mice compared with NE^+/+^ mice in a murine model of the autoimmune disease bullous pemphigoid^33^. It has been reported that the deficiency of circulating AAT is highly associated with lung inflammation and, especially, the early onset of pulmonary emphysema^34^. Additionally, clinical findings have indicated that AAT, which is elevated in the serum of cancer patients, is a serum biomarker for the diagnosis of lung cancer and prostate cancer^35^. Previous studies showed that EGCG inhibits PI3K/Akt/mTOR signaling in various tumor cells^4^,^36^. Our previous data demonstrated that the inhibition of neutrophil elastase-induced cell proliferation using curcumin (a type of polyphenol similar to EGCG) is also dependent on the PI3K pathway in A549 cells^14^. Recently, it was shown that neutrophil elastase released by activated neutrophils within the lung is absorbed by adjacent epithelial tumor cells and degrades IRS-1, which is a binding partner of the p85 regulatory subunit of PI3K, thereby inducing the hyperactivity of the PI3K pathway, which leads to uncontrolled tumor cell proliferation^19^. Our results showed that the neutrophil elastase-induced decrease in IRS-1 expression was significantly inhibited by EGCG in A549 cells. The suppression of Akt and ERK1/2 has been thought to mediate the anti-tumor migration property of EGCG^37^. Therefore, we explored whether EGCG treatment remarkably suppressed neutrophil elastase-induced phosphorylation and activation of PI3K/Akt. Our results showed that the expression of Ser473-phosphorylated Akt was increased in neutrophil elastase-induced A549 cells and was down-regulated after co-treatment with EGCG and neutrophil elastase. Moreover, we observed the phosphorylation of Akt at Thy308. Compared with the regulation of Ser473 phosphorylation, the phosphorylation state of Thy308 was not significantly changed after treatment with neutrophil elastase. These results indicated that the migration-promoting effect of neutrophil elastase was not mediated by Akt phosphorylation at Thy308 but might be mediated by the regulation of p473-Akt. In the positive control group, neither p308-Akt nor p473-Akt expression was significantly changed after treatment with the inhibitor of neutrophil elastase sivelestat sodium. We speculated that sivelestat sodium, as a selective neutrophil elastase inhibitor, cannot suppress the metastasis of lung cancer, in contrast to the multi-targeting natural product EGCG, via the PI3K/Akt signaling pathway.

Taken together, these data suggest that the direct binding of EGCG to neutrophil elastase and the stimulation of AAT by EGCG are crucial for the inhibition of the neutrophil elastase-induced migration of A549 cells by EGCG. However, the molecules and signaling pathways that modulate the expression of AAT remain unclear, and it is important to explore the mechanisms that regulate AAT, which is a SERPIN that is highly related to lung inflammation and lung cancer. Additionally, the role of EGCG in the neutrophil elastase-induced induction of tumor metastasis and growth in other xenograft murine models of lung cancer remains to be investigated.

## Conclusions

**Figure 8:**
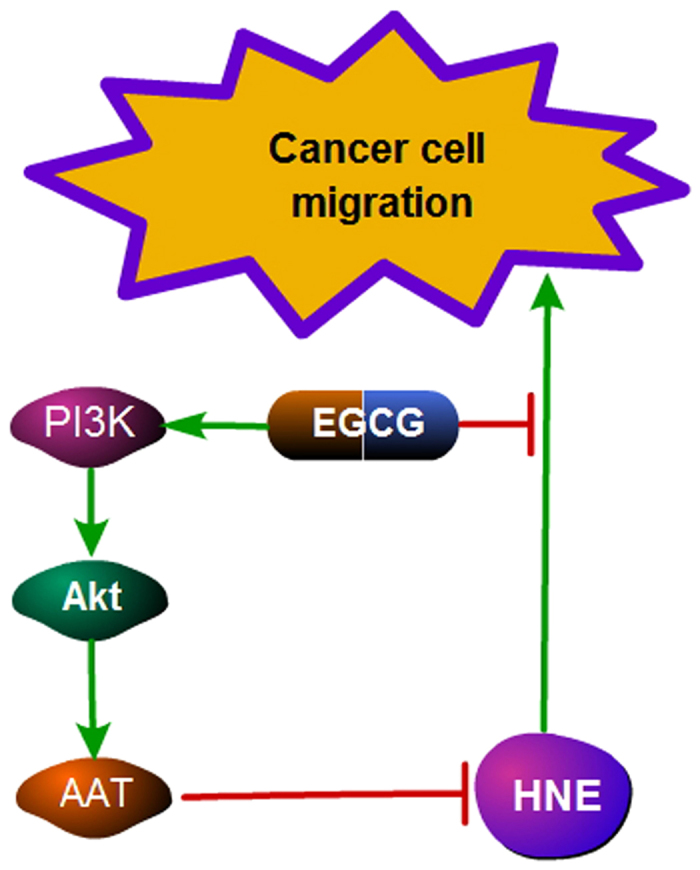
EGCG regulates the neutrophil elastase-induced migration of A549 cells. EGCG inhibits the activity of neutrophil elastase by directly binding to neutrophil elastase. EGCG enhances the expression of AAT by up-regulating the Akt/PI3K signaling pathway.

This study demonstrates that EGCG, an inhibitor of neutrophil elastase, modulates the migration of NSCLC A549 cells and blocks the neutrophil elastase-induced migration of A549 cells by up-regulating AAT expression. These results provide a novel inflammation-related mechanism by which EGCG prevents tumor metastasis and suggests the potential of EGCG for the treatment of other inflammation-related diseases in the lung, such as emphysema (Fig. 8).

## Additional Information

**How to cite this article**: Xiaokaiti, Y. *et al.* EGCG reverses human neutrophil elastase-induced migration in A549 cells by directly binding to HNE and by regulating α1-AT. *Sci. Rep.* **5**, 11494; doi: 10.1038/srep11494 (2015).

## Figures and Tables

**Table 1:** Binding Energy Components Obtained from g_mmpbsa.

Components	∆*E*_*elec*_	∆*E*_*vdw*_	∆*G*_*polar*_	∆*G*_*nonpolar*_	∆*G*_*binding*_
	−165.9 ± 14.3	−60.5 ± 15.0	172.6 ± 20.4	−18.0 ± 1.3	−71.8 ± 12.4

∆*E*_*elec*_: Van der Waal energy; ∆*E*_*vdw*_ Electrostattic energy; ∆*G*_*polar*_: Polar solvation energy; ∆*G*_*nonpolar*_: Non-Polar solvation energy; ∆*G*_*binding*_: Binding energy

## References

[b1] Jemal A. *et al.* Global cancer statistics. CA Cancer J Clin 61, 69–90 (2011). 21296855 10.3322/caac.20107

[b2] Mantovani A. & Balkwill F. Cancer-related inflammation. Nature 454, 436–44 (2008). 18650914 10.1038/nature07205

[b3] Sonoda J. I. *et al.* Green tea catechin, epigallocatechin-3-gallate, attenuates the cell viability of human non-small-cell lung cancer A549 cells via reducing Bcl-xL expression. Exp Ther Med 8, 59–63 (2014). 24944597 10.3892/etm.2014.1719 PMC4061191

[b4] Liu S., Wang X. J. & Cui Y. F. PI3K/AKT/mTOR signaling is involved in (−)-epigallocatechin-3-gallate-induced apoptosis of human pancreatic carcinoma cells. Am J Chin Med 41, 629–42 (2013). 23711146 10.1142/S0192415X13500444

[b5] Yang C. S., Maliakal P. & Meng X. Inhibition of carcinogenesis by tea. Annu Rev Pharmacol Toxicol 42, 25–54 (2002). 11807163 10.1146/annurev.pharmtox.42.082101.154309

[b6] Surh Y. J. Cancer chemoprevention with dietary phytochemicals. Nat Rev Cancer 3, 768–80 (2003). 14570043 10.1038/nrc1189

[b7] Khan N., Afaq F. & Mukhtar H. Targeting multiple signaling pathways by green tea polyphenol (-)-epigallocatechin-3-gallate. Cancer Res 66, 2500–5 (2006). 16510563 10.1158/0008-5472.CAN-05-3636

[b8] Sah J. F., Balasubramanian S. & Rorke E. A. Epigallocatechin-3-gallate inhibits epidermal growth factor receptor signaling pathway. Evidence for direct inhibition of ERK1/2 and AKT kinases. J Biol Chem 279, 12755–62 (2004). 14701854 10.1074/jbc.M312333200

[b9] Gregory A. D. & Houghton A. M. Tumor-associated neutrophils: new targets for cancer therapy. Cancer Res 71, 2411–6 (2011). 21427354 10.1158/0008-5472.CAN-10-2583

[b10] Bellocq A. *et al.* Neutrophil alveolitis in bronchioloalveolar carcinoma: induction by tumor-derived interleukin-8 and relation to clinical outcome. Am J Pathol 152, 83–92 (1998). 9422526 PMC1858104

[b11] Lungarella G., Cavarra E. & Martorana P. A. The dual role of neutrophil elastase in lung destruction and repair. Int J Biochem Cell Biol 40, 1287–96 (2008). 18243764 10.1016/j.biocel.2007.12.008

[b12] Luisetti M. & Seersholm N. Alpha1-antitrypsin deficiency. 1: epidemiology of alpha1-antitrypsin deficiency. Thorax 59, 164–9 (2004). 14760160 10.1136/thorax.2003.006494 PMC1746939

[b13] Sun Z. & Yang P. Role of imbalance between neutrophil elastase and alpha 1-antitrypsin in cancer development and progression. Lancet Oncol 5, 182–90 (2004). 15003202 10.1016/S1470-2045(04)01414-7

[b14] Xu Y. *et al.* Curcumin inhibits tumor proliferation induced by neutrophil elastase through the upregulation of alpha1-antitrypsin in lung cancer. Mol Oncol 6, 405–17 (2012). 22507634 10.1016/j.molonc.2012.03.005 PMC5528353

[b15] Kumari R., Open Source Drug Discovery, C. & Lynn, A. g_mmpbsa--a GROMACS tool for high-throughput MM-PBSA calculations. J Chem Inf Model 54, 1951–62 (2014). 24850022 10.1021/ci500020m

[b16] Chen L. *et al.* Cinanserin is an inhibitor of the 3C-like proteinase of severe acute respiratory syndrome coronavirus and strongly reduces virus replication *in vitro*. J Virol 79, 7095–103 (2005). 15890949 10.1128/JVI.79.11.7095-7103.2005 PMC1112131

[b17] Chen S. *et al.* Severe acute respiratory syndrome coronavirus 3C-like proteinase N terminus is indispensable for proteolytic activity but not for enzyme dimerization. Biochemical and thermodynamic investigation in conjunction with molecular dynamics simulations. J Biol Chem 280, 164–73 (2005). 15507456 10.1074/jbc.M408211200 PMC7982548

[b18] Loser B., Kruse S. O. & Nahrstedt A. Inhibition of neutrophil elastase activity by cinnamic acid derivatives from Cimicifuga racemosa. Planta Med 66, 751–3 (2000). 11199135 10.1055/s-2000-9563

[b19] Houghton A. M. *et al.* Neutrophil elastase-mediated degradation of IRS-1 accelerates lung tumor growth. Nat Med 16, 219–23 (2010). 20081861 10.1038/nm.2084 PMC2821801

[b20] Milligan S. A. *et al.* The green tea polyphenol EGCG potentiates the antiproliferative activity of c-Met and epidermal growth factor receptor inhibitors in non-small cell lung cancer cells. Clin Cancer Res 15, 4885–94 (2009). 19638461 10.1158/1078-0432.CCR-09-0109 PMC4299643

[b21] Mukherjee S., Siddiqui M. A. & Malathi K. Epigallocatechin-3-gallate suppresses proinflammatory cytokines and chemokines induced by Toll-like receptor 9 agonists in prostate cancer cells. J Inflamm Res 7, 89–101 (2014). 24971028 10.2147/JIR.S61365 PMC4070858

[b22] Shim J. H. *et al.* Epigallocatechin gallate suppresses lung cancer cell growth through Ras-GTPase-activating protein SH3 domain-binding protein 1. Cancer Prev Res (Phila) 3, 670–9 (2010). 20424128 10.1158/1940-6207.CAPR-09-0185

[b23] Schramm L. Going Green: The Role of the Green Tea Component EGCG in Chemoprevention. J Carcinog Mutagen 4, 1000142 (2013). 24077764 10.4172/2157-2518.1000142 PMC3783360

[b24] Koshiol J. *et al.* Chronic obstructive pulmonary disease and altered risk of lung cancer in a population-based case-control study. PLoS One 4, e7380 (2009). 19812684 10.1371/journal.pone.0007380 PMC2753644

[b25] Fingleton B. Inflammatory proteinase slips into tumor cells. Nat Med 16, 161–3 (2010). 20134467 10.1038/nm0210-161

[b26] Granot Z. *et al.* Tumor entrained neutrophils inhibit seeding in the premetastatic lung. Cancer Cell 20, 300–14 (2011). 21907922 10.1016/j.ccr.2011.08.012 PMC3172582

[b27] Sulea T., Cui Q. & Purisima E. O. Solvated interaction energy (SIE) for scoring protein-ligand binding affinities. 2. Benchmark in the CSAR-2010 scoring exercise. J Chem Inf Model 51, 2066–81 (2011). 21714553 10.1021/ci2000242

[b28] Alasbahi R. & Melzig M. F. Screening of some Yemeni medicinal plants for inhibitory activity against peptidases. Pharmazie 63, 86–8 (2008). 18271311 10.1055/s-2008-1047849

[b29] Zelvyte I., Sjogren H. O. & Janciauskiene S. Effects of native and cleaved forms of alpha1-antitrypsin on ME 1477 tumor cell functional activity. Cancer Detect Prev 26, 256–65 (2002). 12430630 10.1016/s0361-090x(02)00090-9

[b30] Higashiyama M. & Tateishi R. An evaluation of the prognostic significance of alpha-1-antitrypsin expression in adenocarcinomas of the lung: an immunohistochemical analysis. Br J Cancer 65, 300–2 (1992). 1739634 10.1038/bjc.1992.60 PMC1977730

[b31] Zelvyte I., & Janciauskiene S. alpha1-antitrypsin and its C-terminal fragment attenuate effects of degranulated neutrophil-conditioned medium on lung cancer HCC cells, *in vitro*. Cancer Cell Int 4, 7 (2004). 15555067 10.1186/1475-2867-4-7 PMC539361

[b32] Hopkins P. C., Chang W. S. & Stone S. R. Inhibitory mechanism of serpins. Mobility of the C-terminal region of the reactive-site loop. J Biol Chem 272, 3905–9 (1997). 9020092 10.1074/jbc.272.7.3905

[b33] Liu Z. *et al.* The serpin alpha1-proteinase inhibitor is a critical substrate for gelatinase B/MMP-9 *in vivo*. Cell 102, 647–55 (2000). 11007483 10.1016/s0092-8674(00)00087-8

[b34] Jones S. H. Pulmonary emphysema and alpha1-antitrypsin deficiency. Phys Ther 54, 579–83 (1974). 4214194 10.1093/ptj/54.6.579

[b35] El-Akawi Z. J., Abu-Awad & Khader Y. The importance of alpha-1 antitrypsin (alpha1-AT) and neopterin serum levels in the evaluation of non-small cell lung and prostate cancer patients. Neuro Endocrinol Lett 31, 113–6 (2010). 20150872

[b36] Singh B. N., Shankar S. & Srivastava R. K. Green tea catechin, epigallocatechin-3-gallate (EGCG): mechanisms, perspectives and clinical applications. Biochem Pharmacol 82, 1807–21 (2011). 21827739 10.1016/j.bcp.2011.07.093 PMC4082721

[b37] De Amicis F. *et al.* Epigallocatechin gallate inhibits growth and epithelial-to-mesenchymal transition in human thyroid carcinoma cell lines. J Cell Physiol 228, 2054–62 (2013). 23553645 10.1002/jcp.24372

